# UV and Three Derivative Spectrophotometric Methods for Determination of Ezetimibe in Tablet Formulation

**DOI:** 10.4103/0250-474X.41471

**Published:** 2008

**Authors:** Metreyi Sharma, Deepali V. Mhaske, M. Mahadik, S. S. Kadam, S. R. Dhaneshwar

**Affiliations:** Department of Quality Assurance, Bharati Vidyapeeth University Poona College of Pharmacy, Erandwane, Pune-411 038, India

**Keywords:** UV spectrophotometry, third derivative spectrophotometry, ezetimibe

## Abstract

UV, first, second and third derivative spectrophotometric methods have been developed for the determination of ezetimibe in pharmaceutical formulation. The solutions of standard and sample were prepared in methanol. For the first method, UV spectrophotometry, the quantitative determination of the drug was carried at 233 nm and the linearity range was found to be 6-16 μg/ml. For the first, second and third derivative spectrophotometric methods the drug was determined at 259.5 nm, 269 nm and 248 nm with the linearity ranges 4-14 μg/ml, 4-14 μg/ml and 4-16 μg/ml. The calibration graphs constructed at their wavelength of determination were found to be linear for UV and derivative spectrophotometric methods. All the proposed methods have been extensively validated. The described methods can be readily utilized for the analysis of pharmaceutical formulation. There was no significant difference between the performance of the proposed methods regarding the mean values and standard deviations.

The chemical formula of ezetimibe is (3 *R*,4S)-1-(4-fluorophenyl)-3-((3 *S*)-3-(4-fluorophenyl)-3-(hydroxypropyl)-4-(4-hydroxyphenyl)-2-azetidinone^1^. Ezetimibe is the first drug in clinical practice in the class of selective cholesterol uptake inhibitors for the treatment of hyperlipoprotienemia^2^.

Literature survey revealed that a reverse-phase HPLC method has been developed for the determination of ezetimibe in pharmaceutical dosage forms^3^. Liquid chromatographic-tandem mass spectrometric (LC-MS/MS) methods have been developed for the estimation of ezetimibe in biological fluids^4^–^6^. There are no UV and derivative spectrophotometric methods reported for the analysis of ezetimibe in pharmaceutical formulation. Derivative spectrophotometry is an analytical technique of great utility for extracting both qualitative and quantitative information from spectra composed of unresolved bands, and for eliminating the effect of baseline shifts and baseline tilts. It consists of calculating and plotting one of the mathematical derivatives of a spectral curve. Derivative spectrophotometry is now a reasonably prized standard feature of modern micro-computerized UVspectrophotometry^7^. The aim of this work was to develop and validate UV, first, second and third derivative spectrophotometric methods for the determination of ezetimibe in tablet formulation.

Pharmaceutical grade ezetimibe was a generous gift from Ind-swift laboratories Limited (Punjab, India). All analytical grade chemicals were purchased from Merck (India). Ezetimibe tablets (EZTIM, Glenmark Pharmaceutical Ltd., Mumbai, India) containing 10 mg of ezetimibe per tablet was assayed. A Jasco V-530 version UV/Vis spectrophotometer with data processing system was used. UV and derivative spectra of the solutions were recorded in 1 cm quartz cells at a scan speed of 1000 nm/min, a scan range of 200-400 nm for UV, 220-320 nm for first and second derivative and 225-325 nm for third derivative with fixed slit width of 2 nm and data pitch of 0.5 nm.

Stock standard solution was prepared by dissolving 10 mg of ezetimibe in 10 ml methanol. The standard solutions were prepared by dilution of the stock solution with methanol in a concentration range of 6-16 μg/ml for UV, 4-14 μg/ml for first and second and 4-16 μg/ml for third derivative spectrophotometric methods, respectively.

A total of 10 tablets of ezetimibe were accurately weighed and powdered. An amount of tablet triturate equivalent to label claim of ezetimibe was weighed and transferred in 10 ml calibrated volumetric flask, diluted with methanol stirred for about 10 min and then volume made up with methanol. This solution was filtered to remove any insoluble matter. The filtrate was collected in a clean flask. Appropriate dilutions were made to obtain 10 μg/ml with methanol from stock solution for UV and derivative spectrophotometric methods at 259.5, 269 and 248 nm, respectively.

Under the experimental conditions described the graph obtained for UV, first, second and third derivative spectra showed linear relationship. Regression analysis using the method of least-squares was made for the slope, intercept and correlation coefficient values. The regression equations of calibration curves were y = 4.415×10^-2^ x+2.077×10^-2^, (r = 0.9996) for the UV, y = 1.362×10^-3^ x+5.890×10^-4^, (r = 0.9998) for the first, y = 4.477×10^-5^ x+3.772×10^-5^, (r = 0.9998) for the second and y = 4.648×10^-6^ x-1.082×10^-6^, (r = 0.9999) for the third derivative spectrophotometric methods, respectively. The range was found to be 6-16 μg/ml for UV, 4-14 μg/ml for first and second derivative and 4-16 μg/ml for third derivative spectrophotometric methods. The statistical parameters given are the regression equation calculated from the calibration graphs, along with the standard deviations of the slope (S_b_) and intercept (S_a_) on the ordinate. The results are presented in Table 1.

**TABLE 1:** STATISTICAL DATA FOR CALIBRATION CURVES FOR DETERMINATION OF EZETIMIBE

Parameters	UV	First derivative	Second derivative	Third derivative
Range (μg/ml)	6-16	4-14	4-14	4-16
Slope (b)	4.415×10^-2^	1.362×10^-3^	4.477×10^-5^	4.648×10^-6^
Standard deviation of slope (Sb)	6.920×10^-4^	1.329×10^-5^	7.320×10^-7^	1.143×10^-7^
Intercept (a)	2.077×10^-2^	5.890×10^-4^	3.772×10^-5^	-1.082×10^-6^
Standard Deviation of intercept (Sa)	1.557×10^-2^	1.7×10^-4^	4.880×10^-6^	4.861×10^-6^
Correlation coefficient (r)	0.9996	0.9998	0.9998	0.9999
LOD (μg/ml)	0.36	0.23	0.21	0.15
LOQ (μg/ml)	1.09	0.69	0.63	0.46

LOD is limit of detection and LOQ is the limit of quantitation, n=6

First, second and third derivative spectra of ezetimibe in standard and drug formulation solutions showed that the wavelength of maximum absorbance did not change. Comparison of the zero-order spectrum with first and second derivative spectrum of ezetimibe in standard and drug formulation solutions showed that the wavelength of maximum absorbance did not change. According to the results obtained by recovery study, the derivative spectrophotometric method is able to access the analyte in presence of excipients and hence, it can be considered specific.

Limit of detection (LOD) and limit of quantitation (LOQ) were determined by using the formula based on the standard deviation of response and the slope The limit of detection (LOD) and limit of quantification (LOQ) were calculated by using the equations LOD = 3×σ/S and LOQ = 10×σ/S, where σ is the standard deviation of intercept, S is the slope (Table 1).

To study the accuracy of the proposed methods, and to check the interference from excipients used in the dosage forms, recovery experiments were carried out by the standard addition method. This study was performed by addition of known amounts of ezetimibe to preanalyzed solutions of commercial tablets. The mean recoveries were found to be 98.45-100.85, 98.07-99.83, 100.13-100.70, 100.49-101.46 % respectively for UV, first, second, and third derivative spectroscopy.

To determine the precision of the method, ezetimibe solutions at a concentration of 6, 8, 10 μg/ml were analyzed each in triplicate. Solutions for the standard curves were prepared fresh everyday. The methods were found to be precise. The % RSD values for intra day precision studies were found to be 0.49, 0.49, 0.60 and 0.47 for UV, first, second and third derivative spectroscopy. The % RSD values for inter day precision studies were found to be 0.61, 0.60, 0.33 and 0.93 for UV, first, second and third derivative spectroscopy, respectively.

For robustness and ruggedness of analytical methods the tests mentioned below were carried out. The robustness of developed methods was tested by changing parameters such as degree of derivation, wavelength range and N value and the optimum parameters were chosen for this study. The UV and derivative spectrophotometric determinations of ezetimibe were carried out by two different analysts on the same instrument with the same standard. The results showed no statistical differences suggesting that the developed methods were robust and rugged (Table 2). The developed methods are accurate, sensitive and precise and can be easily applied to the pharmaceutical formulation.

**TABLE 2:** RESULTS OF ANALYSIS OF EZITIM TABLETS

Statistical value	UV	First derivative	Second derivative	Third derivative
X*	10.17	9.90	9.97	10.03
SD	0.08	0.04	0.07	0.06

X* mean of six readings, SD is the standard deviation. Ezitem are tablets containing 10 mg of ezetimibe, n=6
